# Optimization of a Protocol for Launching Grapevine Infection with the Biologically Active cDNA Clones of a Virus

**DOI:** 10.3390/pathogens12111314

**Published:** 2023-11-03

**Authors:** Mehdi Shabanian, Caihong Li, Ali Ebadi, Valerian Dolja, Baozhong Meng

**Affiliations:** 1 Department of Molecular and Cellular Biology, University of Guelph, Guelph, ON N1G 2W1, Canada; caihong@uoguelph.ca (C.L.); bmeng@uoguelph.ca (B.M.); 2 Department of Horticulture, College of Agriculture and Natural Resources, University of Tehran, Karaj 31587-11167, Iran; aebadi@ut.ac.ir; 3 Department of Botany and Plant Pathology, Oregon State University, Corvallis, OR 97331, USA; doljav@oregonstate.edu

**Keywords:** grapevine leafroll disease, *Closteroviridae*, GLRaV-3 cDNA clones, RT-qPCR, Western blot, vacuum agro-infiltration, agro-pricking, agro-drenching, agro-injection, Koch’s postulates

## Abstract

Grapevine leafroll disease (GLRD) is the most globally prevalent and destructive disease complex responsible for significant reductions in grape yield and quality as well as wine production. GLRD is associated with several positive-strand RNA viruses of the family *Closteroviridae*, designated as grapevine leafroll-associated viruses (GLRaVs). However, the specific etiological role of any of these GLRaVs in GLRD has not been demonstrated. Even though GLRaV-3 is considered the chief GLRD agent, little is known about the molecular, cellular, and pathological properties of this virus. Such a knowledge gap is due to multiple factors, including the unavailability of biologically active virus cDNA clones and the lack of reliable experimental systems for launching grapevine infection using such clones. In this work, we tested four methods for inoculating tissue-cultured grapevine plantlets with cDNA clones of GLRaV-3: (i) vacuum agro-infiltration; (ii) agro-pricking; (iii) agro-drenching; and (iv) agro-injection. We showed that vacuum agro-infiltration was the most effective of these methods. Furthermore, we examined the impacts of different experimental conditions on the survival and infectivity rate of grapevines after infiltration. To verify the infectivity rate for different treatments, we used RT-PCR, RT-qPCR, and Western blotting. We found that humidity plays a critical role in the survival of plantlets after agro-infiltration and that the use of RNA silencing suppressor and dormancy treatment both had strong effects on the infection rates. To our knowledge, the experimental protocol reported herein is the most effective system for launching the infection of grapevine using cDNA clones of grapevine viruses featuring up to a 70% infection rate. This system has strong potential to facilitate grapevine virology research including the fulfillment of Koch’s postulates for GLRD and other major virus diseases as well as identifying the molecular, cellular, and pathological properties of GLRaVs and, potentially, other important grapevine viruses.

## 1. Introduction

To date, 86 distinct viruses belonging to 17 families and 34 genera have been demonstrated to infect grapevine (*Vitis* spp.), the largest number of viruses detected in a particular plant species [1,2,3,4]. The occurrence of so many different viruses in grapevine could be due to the long history of grapevine domestication and exposure to the existing viruses or the high incidence of persistently infecting viruses. This list can be extended to include the extensive exchange of grapevine germplasm between grape-growing countries or regions, the broad use of grafting as an essential viticulture practice [5], and the substantial research effort aimed toward identifying viruses infecting this high-cash-value crop using new detection technologies such as next-generation sequencing (NGS) [3,6]. Grapevines are commonly infected with multiple viruses, likely because unregulated grafting has permitted the broad spread of viruses. Because of these common co-infections, it is difficult to establish the exact etiological roles of a given virus in a disease. All grapevine viruses are efficiently disseminated through infected propagating materials and grafting between varieties of scions and rootstocks [7], while many of these viruses can also be spread by different invertebrate vectors.

Currently, most of the documented grapevine viruses are classified into four major groups, which are associated with four disease complexes. One group of viruses belongs to the family *Secoviridae* and is associated with infectious degeneration/decline. *Grapevine fanleaf virus* (GFLV), and the fanleaf degeneration caused by GFLV, is the oldest known and one of the most widespread viral diseases of grapevine [7]. The second group of viruses is associated with the rugose wood (RW) complex and belongs to the family *Betafexiviridae*. This group includes *Grapevine rupestris stem pitting-associated virus* (GRSPaV), Grapevine *virus A* (GVA), and *Grapevine virus B* (GVB), among other related viruses [1,7]. The third group of viruses such as *Grapevine fleck virus* (GFKV) belongs to the family *Tymoviridae,* which is associated with the fleck disease complex [1,7]. Finally, the fourth group of viruses belongs to the family *Closteroviridae*, which is associated with the grapevine leafroll disease (GLRD) complex [1,8]. In recent years, a few new important diseases such as grapevine red blotch, caused by theDNA virus ‘*Grapevine red blotch virus*’ (GRBV) and grapevine leaf mottling and deformation caused by *Grapevine Pinot gris virus* (GPGV) have been identified around the world [9,10,11,12,13].

Among all these diseases, GLRD is inarguably the most important viral disease in grapevines economically, comparable to those caused by fungal pathogens. The GLRD complex occurs in all major grape-growing regions worldwide, causing significant losses to the grape and wine industry. Grapevine infections with GLRD result in major reductions in yield and fruit quality and shorten the productive lifespan of vineyards. Although GLRD is associated with six species of grapevine-leafroll-associated viruses (GLRaVs) belonging to three genera of the *Closteroviridae* family, *Grapevine leafroll-associated virus 3* (GLRaV-3) from the *Ampelovirus* genus is regarded as the most economically destructive of the GLRaVs due to its global distribution, high prevalence, and disease severity [14,15,16].

To investigate the molecular or cellular aspects of RNA virus infection or virus gene functions, it is essential to generate experimentally amenable, biologically active cDNA clones of the virus genomes. These cDNA clones can initiate virus infection either upon mechanical inoculation of the susceptible plants or upon agro-infiltration [17,18]. The latter approach is especially critical for non-mechanically transmissible viruses. Recently, cDNA clones of several grapevine viruses have been developed and utilized as basic tools in molecular virology [19,20,21,22,23,24,25,26]. In addition to the application of reverse genetics for studying virus and host functional genomics, virus cDNA clones are critical for fulfilling Koch’s postulates for virus diseases.

Unlike many herbaceous plants that can be readily infected via mechanical inoculation with cloned virus cDNA, grapevine and other woody perennials are recalcitrant toward mechanical inoculation. An alternative to mechanical inoculation is agro-infection, which relies on the natural ability of *Agrobacterium tumefaciens* to deliver target DNA into the nuclei of plant cells. Agro-infection can be achieved using four available inoculation methods, depending on the virus–host combination. In the first method, an agrobacterium suspension is directly rubbed on the leaf surface or injected into the leaves using a needleless syringe. This method is suitable for the viruses of herbaceous hosts [17,18,27,28]. The second method is agro-drenching, which has been effective for the *Solanaceae* species via the drenching of soil with agrobacterium suspensions [29]. Agro-drenching was shown to also work for some grapevine viruses such as *Grapevine virus A* (GVA) and *Grapevine rupestris stem-pitting-associated virus* (GRSPaV) [20,22]. However, this method requires a long time to establish virus infection and achieves a very low infection rate. The third method is agro-pricking, which was previously described and used by Yepes et al. [24] in the agro-infection of grapevine plantlets using the infectious clones of *Grapevine red blotch virus* (GRBV). Based on their report, this method worked well as it resulted in a good infection rate. The fourth, more efficient method is vacuum-mediated agro-infiltration developed for woody plant viruses such as *Grapevine leafroll-associated virus-2*(GLRaV-2), *Apple chlorotic leaf spot virus* (ACLSV), GRBV, and *Grapevine Pinot gris virus* (GPGV) [21,22,26,30].

Despite substantial progress in developing methods for the delivery of cDNA clones, further improvements of these methods remain crucial for empowering the research and practical applications of the viruses of grapevine and other woody plants. Accordingly, the main objective of this study was to optimize an experimental system for launching grapevine infections with virus cDNA clones in general and GLRaV-3 in particular. We first compared the performance of the four mentioned inoculation methods in initiating grapevine infection with GLRaV-3 cDNA clones and found that vacuum-mediated agro-infiltration is the most effective of these. We further tested a series of experimental conditions impacting the survival of the infiltrated plantlets and infectivity rate and developed the optimized protocol for the entire process of vacuum agro-infiltration and the subsequent plant recovery. This protocol has a strong potential for broad applications in woody plant virology, including the fulfillment of Koch’s postulates, virus–host interactions including pathogenesis, as well as virus and plant functional genomics using virus vectors for gene expression and virus-induced RNA interference.

## 2. Material and Methods

### 2.1. Establishment of Grapevine Tissue Culture

**Figure 1:**
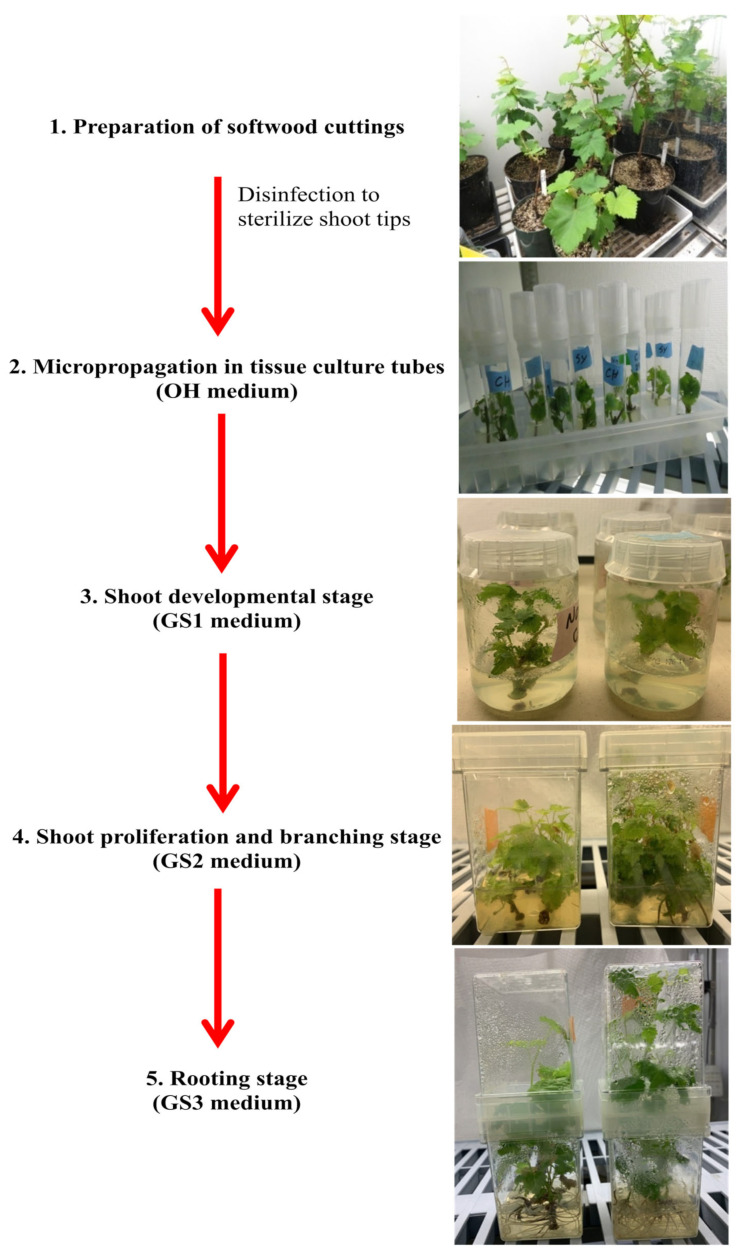
Establishment of grapevine tissue culture from grapevine cuttings. This involved five stages: (1) preparation of softwood cuttings; (2) micro-propagation of shoot tips in glass tubes; (3) shoot development; (4) shoot proliferation and elongation; and (5) rooting. The entire process took approximately 4 months.

The first step was to generate grapevine plantlets from certified virus-free grapevine mother plants via tissue culture. In this study, certified cuttings from different varieties including Cabernet franc, Syrah, and Chardonnay, kindly provided by Foundation Plant Services US-Davis, were rooted and potted and later used as mother plants to establish the tissue culture system. Plants established in the greenhouse were first tested for the most common grapevine viruses involved in important diseases in commercial grapes, such as leafroll, rugose wood, infectious degeneration and decline, and newly identified viruses such as red blotch. To reach this goal, the plants were monitored for disease symptoms and tested using multiplex RT-PCR with primers targeting 17 grapevine viruses, as described by Xiao et al. [31]. Once established, grapevine plants in the greenhouse produced a good number of shoots. The new shoots were cut and treated for growth on an agar medium. For this reason, the method of semi-sterilized tissue culture for the rapid propagation of grapevines used by Shan and Seaton [32] was adopted with some changes. First, all leaves were removed, and shoot tips, roughly 7–10 cm in length, with one node on each were cut. These cuttings were rinsed for 1 h under running tap water and then surface-sterilized for 6 min in a 5% bleach solution while stirring. From this point onward, all plant work was conducted in a laminar flow hood. After sterilization, all the single-node cuttings were rinsed three times in sterile deionized water for 10 min each. Subsequently, all the cuttings were drained on sterile tissue paper. Finally, the bases of the single-node cuttings were cut off with a sterile blade at a 45-degree angle and planted into glass tubes with OH medium, the shoot initiation agar medium containing 0.2 g/L of cefotaxime (Table S1). One single-node cutting per tube was maintained in the growth chamber at a constant temperature of 22 °C with a 16 h photoperiod at a light intensity of 50 µmol/m^2^/s. These tubes were periodically checked for contamination. After approximately 2 weeks, once the new shoots started growing, a GS1 liquid medium containing 0.2 g/L of cefotaxime was added to the glass tubes (Table S1). Approximately 2–3 weeks later, the new shoots were removed from the stems and transferred into new containers with a GS1 agar medium. New shoots established on the agar media moved through the three typical grape stages: GS1, GS2, and GS3 (Table S1). While the GS1 medium allowed plant growth without promoting shoot or root elongation, the GS2 medium promoted shoot proliferation and elongation, and the GS3 medium promoted root development (Figure 1).

### 2.2. Agrobacterium Preparation

Agro-infiltration was conducted using *A. tumefaciens* (EHA105) containing the binary vector pCB301 carrying a full-length GLRaV-3 cDNA clone (which we designate as pLR3 from here on) or its GFP-tagged variant (which we designate as pLR3-GFP), which were recently constructed in our lab (data not published), and vLR2-p24 (GLRaV-2 RNA silencing suppressor) (RSS) was used [21]. Briefly, after streaking glycerol stocks of the agrobacterium on LB agar plates containing kanamycin (50 μg/mL) and rifampicin (25 μg/mL) and incubating at 30 °C for 2 days, a single colony was cultured in 5 mL of LB containing the same antibiotics and 10 mM of morpholine ethanesulfonic acid (MES) (pH 5.85) and 20 μM of acetosyringone at 30 °C with shaking at 250 rpm. The overnight cultures were sub-cultured and grown at 30 °C with shaking in a larger volume of the same LB medium until reaching an optical density at 600 nm (OD_600_) of 1.0. Then, the agrobacterial cultures were centrifuged for 10 min at 5000 rpm, and the resultant pellets were re-suspended and washed in an infiltration buffer (10 mM of MES (pH 5.85), 10 mM of MgCl_2_, and 150 μM of acetosyringone). A second round of centrifugation was carried out, and the pellets were re-suspended in an appropriate amount of the infiltration buffer. The final OD_600_ of the agrobacterial suspension was adjusted to 2.0 for infectious viral clones or to 0.5 for vLR2-p24, followed by induction at room temperature for two hours [21]. 

### 2.3. Four Methods Applied in Agro-Inoculation

**Figure 2:**
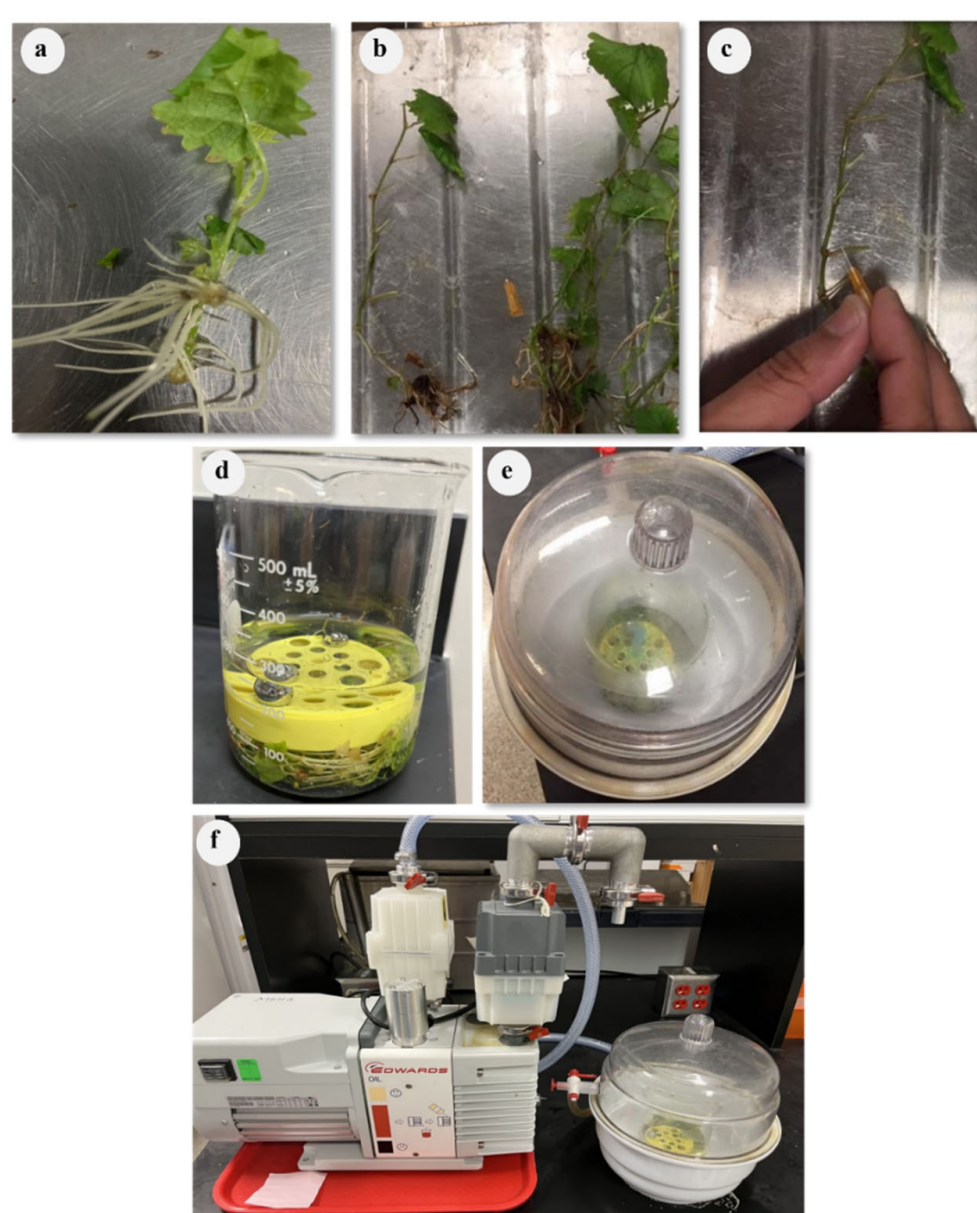
Preparation of grapevine plantlets and vacuum-mediated agro-infiltration. Grapevine plantlets, 8–11-week-old, generated from tissue culture were removed from magenta boxes and placed on a bench (**a**), trimmed with a pair of scissors to remove some of the leaves and roots (**b**), and pierced with a 31-gauge needle to create minor wounds along the stem and major roots (**c**).After trimming, plantlets were placed in a 500 mL beaker containing 300 mL of agrobacterial suspension carrying viral full-length clone (**d**), which was subsequently placed in a nucerite desiccator (**e**) that was connected to a vacuum pump (**f**).

Four different techniques including vacuum-based agro-infiltration, agro-pricking, agro-drenching, and agro-injection were tested and compared in this study. In all methods, 8–11-week-old, healthy-looking, tissue-cultured grapevine plantlets were used. To prepare them for agro-inoculation, the plantlets were trimmed with a pair of scissors to remove most of the leaves and side shoots, especially near the bottom of the plantlets. In addition, some of the roots were trimmed to one-third of their length. In the vacuum infiltration and pricking methods, trimmed plantlets were poked on their stems and roots with a 31-gauge needle (Figure 2a–c). The reason behind the trimming and pricking was to create minor wounds to facilitate the entry of the agrobacterium into the plant tissue. Trimming also reduced the number of leaves and branches to allow better recovery from the shock produced by the infiltration process and rapid loss of humidity after transplanting them into soil after infiltration.

#### 2.3.1. Vacuum-Based Agro-Infiltration

To examine the infectivity of our pLR3, we followed the protocol of Kurth et al. [21] with minor modifications. The agrobacterium preparation was performed, as mentioned earlier, for all agrobacterium containing viral clones including pLR3, pLR3-GFP, or p24 as an RSS. In each beaker, 300 mL of each virus genome containing the agrobacterial suspension were prepared at anOD_600_ of 2.0 for the viral clone and OD_600_ of 0.5 for the RSS p24. 

**Figure 3:**
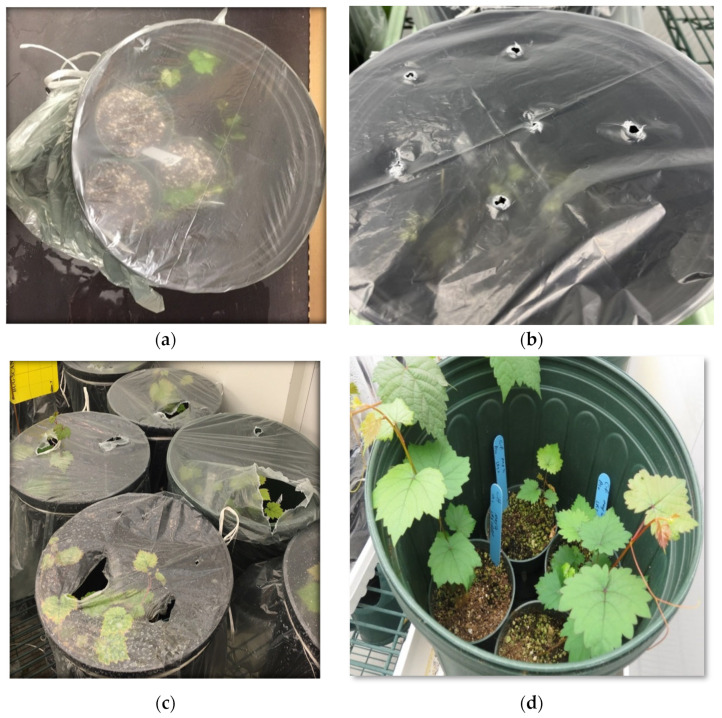
Transplantation, recovery, and establishment of grapevine plantlets in soil. Infiltrated plantlets were gently transplanted individually into 2.5-inch round plastic pots, several of which were then placed in 3-gallon plastic pots followed by covering with transparent plastic sheets (**a**). These covered pots were kept in a growth room at 21–22 °C with a 16/8 h photoperiod for 3–4 weeks. To gradually expose the plantlets to the conditions in the growth room, small holes were poked using a pen on each plastic cover after 2–3 weeks post-infiltration (**b**). These holes were gradually enlarged to allow more air exchange (**c**). When the plantlets were fully established in the soil, the plastic covers were removed (**d**).

The infiltration procedure comprised three cycles, each including applying vacuum treatment for a desired duration, followed by a quick vacuum release. After the infiltration procedure, the plantlets were rinsed with tap water and gently potted in 2.5-inch round plastic nursery pots and maintained in the growth room. To maintain the humidity, the pots containing infiltrated plantlets were placed in 3-gallon plastic pots covered with clear plastic sheets and allowed to recover in a growth chamber for one month at a temperature of 21–22 °C with a 16 h photoperiod at a light intensity of 50 µmol/m^2^/s (Figure 3a).

#### 2.3.2. Agro-Pricking

In the second method, the protocol described by Yepes et al. [24] with some modifications was applied. After trimming the plantlets, the stems and roots were again gently wounded by pricking them with a 31-gauge needle dipped in the agar culture of *A. tumefaciens* containing viral infectious clones, followed by submerging them for 30 min in a beaker with 300 mL of each viral-containing agrobacterial cells as described for method 1. Afterward, they were washed, potted, and kept in the conditions described previously.

#### 2.3.3. Agro-Drenching

Agro-drenching was conducted as described by Ryu et al. [29] with minor changes. First, grapevine plantlets were trimmed, poked with a 31-gauge needle as described above, and planted in 2.5-inch round plastic nursery pots. Afterward, 5–10 mL of the agrobacterial suspension at the same optical density, as described above, was poured into the soil close to the crown of each plantlet. These mini pots were kept in the growth chamber underthe same conditions described earlier. 

#### 2.3.4. Agro-Injection

Similar to the other methods, 8–11-week-old tissue-cultured plantlets were first trimmed, followed by injection with an agrobacterial suspension containing the viral clone alone or a mixture of agrobacteria containing both the viral clone and the construct expressing p24. For this procedure, the bacterial inoculum was diluted with infiltration buffer to reach the final OD_600_ of 1.0. The bacterial suspensions were gently injected using a 1 mL needleless syringe through the stomata of the lower epidermis [33].

It should be noted that in all four treatments, grapevine plantlets were mock-infiltrated using the respective method with an agrobacterium containing the p24 expression plasmid only.

### 2.4. The Effects of Various Factors on the Survival and Infectivity Rate Using Vacuum-Based Agro-Infiltration Technique

After conducting all four methods and comparing the preliminary results of nested-RT-PCR and RT-qPCR, based on the infectibility percentages and virus expression levels in the plantlets, we found that vacuum-based agro-infiltration gave the best performance. Therefore, we decided to focus only on this technique to test different factors and conditions to optimize this experimental system to launch the infection of grapevine using infectious viral clones. It is important to note that in each experiment, only one factor was varied at a time, and all other conditions were kept constant.

#### 2.4.1. Age and the Cultivar of Tissue-Cultured Plantlets

To study the impacts of the age of plantlets on the survival and infection rate, three groups of plantlets were chosen. The first group was 5–7 weeks old, the second group was 8–11 weeks old, and the third group was 12–16 weeks old. Three grapevine cultivars were tested (Cabernet franc, Syrah, and Chardonnay) to find out which cultivar is more conducive to the vacuum infiltration procedure, as judged by both the survival and infection rate. In this experiment, the pLR3-containing agrobacterium at an OD_600_ of 2.0 and p24 at an OD_600_ of 0.5 was infiltrated into the plantlets with three vacuum cycles of 10 min for each, followed by a quick vacuum release. The infiltrated plantlets were monitored for survival and assayed for infectivity at 2 months post-infection.

#### 2.4.2. Impacts of Humidity Level

The humidity level is one of the most important factors in a plant growth chamber, as it affects the rate of transpiration and nutrient absorption. To study the impacts of the humidity level on the survival and infection rates of grapevine plantlets post-agro-infiltration, the plastic covers of the pots were removed from the pots containing infiltrated plantlets at three different times in a gradual or instant manner. Herein, the pLR3-containing agrobacterium at an OD_600_ of 2.0 and p24 at anOD_600_ of 0.5 was infiltrated into the Syrah and Cabernet franc plantlets following the standard procedure, as described previously. After infiltration and potting the plantlets in 2.5-inch round plastic nursery pots, these small pots were placed in 3-gallon plastic pots, which were covered with plastic sheets and kept in a growth chamber, and one week later, the plastic covers were removed instantly or gradually (Figure 3a,b). In the gradual manner, after one-week post-infiltration, several holes with a diameter of 4–5 mm were made by poking the plastic cover. These holes were gradually made bigger until the covers were removed (Figure 3c,d).In the other pots, the covers were removed after 2 or 3weeks either gradually or instantly to observe the effects of humidity on both the survival and the infection rates. 

#### 2.4.3. Effects of Vacuum Duration and Agrobacterial Density (OD_600_) on Plantlet Survival and Infectivity

As the vacuum treatment is an important step in this infiltration procedure, the effect of various vacuum durations on the survival and infection rates was tested. Three-time durations were evaluated, 5, 10, and 15 min, for three cycles each. For example, consider the 5 min duration level. Plantlets that were submerged in the agrobacterial suspension in a beaker were subjected to vacuum treatment for 5 min, followed by a quick release. The same procedure was repeated twice. In the second part of this experiment, various OD_600_ values for the pLR3-containing bacterium, including 1.0, 2.0, and 3.0, and different OD_600_ values for the supplemental bacterium containing the RSS p24, such as 0.5 and 1.0, were infiltrated into 8–11-week-old grapevine plantlets with three vacuum cycles of 10 min each and a quick pressure release after each cycle.

#### 2.4.4. Effects of Various RSSs on Infectivity

To investigate the impacts of viral RNA-silencing suppressors (RSSs) on the infection rates, several such suppressors derived from different viruses including p24 of GLRaV-2, p19 of *Tomato bushy stunt virus*, p21 of *Beet yellows virus*, HC-Pro of *Turnip mosaic virus*, and TCV-CP of *Turnip crinkle virus* were tested. As negative controls, plantlets were vacuum-infiltrated with an agrobacterium containing only pLR3 without any RSS. In this experiment, a cDNA-clone-containing agrobacterium at an OD_600_ of 2.0 and different supplemental agrobacteria containing RSSs at an OD_600_ of 1.0 were infiltrated into 8–11-week-old grapevine plantlets with a vacuum duration of 10 min for each cycle. 

#### 2.4.5. Impact of Dormancy Treatment on the Infection Rates and Viral Titer

To study the influence of dormancy on the infectivity rate, viral titer, and symptom development, grapevine plants that were inoculated via agro-infiltration 8–10 months earlier were subjected to either one or two cycles of dormancy. As negative controls, grapevines derived from the same agro-infiltration procedure were kept under normal growth conditions. Each dormancy treatment comprised 50 grapevines, 25 of which tested positive, whereas the remaining 25 plants tested negative for GLRaV-3 based on nested RT-PCR. The dormancy treatment was repeated once for all experiments. Before inducing dormancy, each plant was trimmed, such that only three to four leaves were left. In the single dormancy treatment, 50 grapevine plantlets were subjected to an incremental drop in temperature over a 3-week period. In week one, the temperature was reduced from 22 to 16 °C; in week two, the temperature was further dropped to 10 °C; and from the third week onward, the plants were kept at 4 °C for 2 months. At the end of the cold treatment cycle, the temperature was returned to 22 °C using the same increment but in reverse order. 

The double dormancy treatment simply comprised two cycles of the single dormancy treatment that were interrupted by a 2-month break period between them. In other words, at the completion of the first dormancy cycle, plants were returned to 22 °C for 2 months to recover and grow followed by a second cycle of dormancy.

### 2.5. Infectivity Assays

**Figure 4:**
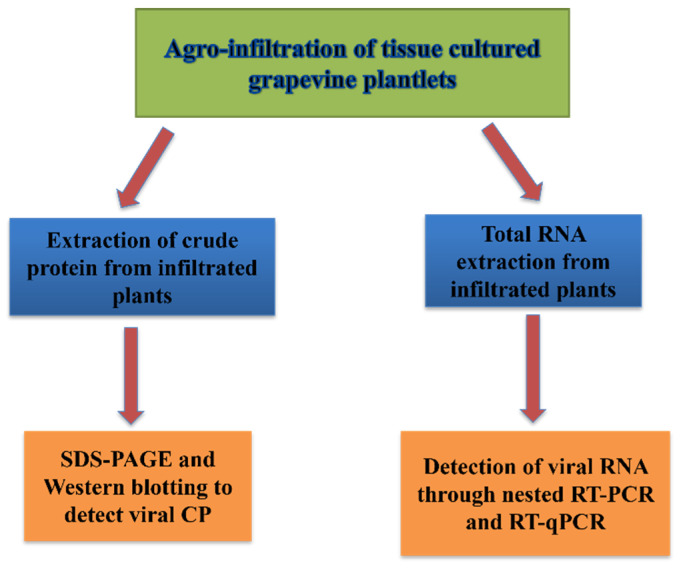
A schematic overview of different methods that were used to demonstrate infectivity in grapevines after infiltration with viral infectious clone. CP: viral capsid protein; RT-PCR: reverse transcription–polymerase chain reaction; qPCR: quantitative PCR; SDS-PAGE: sodium dodecyl sulfate–poly acrylamide gel electrophoresis.

Different methods were used to detect the virus after infiltration. The best and most sensitive method, especially in the early months post-infiltration, was based on the detection of virus RNA, such as nested RT-PCR and RT-qPCR. Other assays such as sodium dodecylsulfate–polyacrylamide gel electrophoresis (SDS-PAGE) and Western blotting, which are based on the expression of the virus capsid protein, were also helpful for testing the infectivity success but were slightly less sensitive. A schematic of these methods is shown in Figure 4.

#### 2.5.1. Total RNA Isolation, RT-PCR, Nested RT-PCR, and RT-qPCR

To test for the systemic infection of the virus resulting from agro-infiltration, new and non-infiltrated leaves of grapevines were collected at two months post-infiltration (mpi). Subsequently, the veins and petioles were obtained from the leaf samples and ground to fine powders in liquid nitrogen using a mortar and pestle and stored at −80 °C. Total RNA was extracted from 50 mg of the ground tissue samples following the protocol described by Xiao et al. [34]. The quality and concentration of RNA preps were measured with a Nano Drop (ND-1000, Thermo Fisher, Mississauga, ON, Canada) and stored at −80 °C until further use. First-strand cDNA synthesis was primed with random hexamers using the High-Capacity cDNA Reverse Transcription Kit (Thermo Fisher, Mississauga, ON, Canada), essentially following the protocol of Shabanian et al. [31].

Conventional RT-PCR was conducted by using virus-specific primers following Shabanian et al. [35], with a minor change in the cDNA used in PCR. Because of the extremely low level of viral RNA present in infiltrated plants, in each PCR reaction, 2–3 μL of cDNA was used. Furthermore, nested RT-PCR and RT-qPCR were used to detect the low level of virus RNA. Herein, two sets of primers with the same reverse primer were used to amplify the region at the 3′ end of the GLRaV-3 genome followed by electrophoresis on agarose gels. The first round of the nested PCR reaction mix (25 µL) contained 2–3 µL of cDNA, 2.5 µL of 10× PCR buffer (containing 2.0 mM of MgCl_2_), 0.2 mM of dNTPs, 0.2 µM of each primer (F1_16289 and R_17240), and 1.0 unit of Taq DNA polymerase (Gene DireX, Taoyuan, Taiwan) to amplify a 952 bp DNA fragment. The PCR conditions included an initial denaturation step at 94 °C for 5 min, then 35 cycles at 94 °C for 30 s, 53 °C for 30 s, and 72 °C for 1 min, followed by a final extension at 72 °C for 7 min. In the second round of PCR, everything remained the same as in the first round of PCR except the following: (1) 2 µL of the amplification product from the first round of PCR was used as a template instead of cDNA; (2) an internal forward primer (F2_16896) and the same reverse primer were used to amplify a 345 bp DNA fragment; and (3) PCR was conducted for 20 cycles instead of 35 cycles. The primers used for these assays are shown in Table S2.

#### 2.5.2. Western Blotting

Western blot was performed following the protocol of Shabanian et al. [35] with some modifications. As was mentioned earlier, the viral titer in infiltrated plants was extremely low, so to obtain more reliable results, petioles and leaf-midribs were cut using a single-edged razor blade and ground to a fine powder in liquid nitrogen. Subsequently, 0.5 g of the tissue powder was homogenized in four volumes of a protein extraction buffer (200 mM of Tris-Cl (pH 8.2), 140 mM of NaCl, 500 mM of polyvinylpyrrolidone [PVP]-40, 10 mM of β-mercapto-ethanol, and 1 mM of phenylmethyl-sulfonyl fluoride). In this experiment, a primary antibody generated in rats against the recombinant CP of GLRaV-3 at a dilution of 1:5000 and a secondary antibody (goat anti-rat IgG) conjugated with horseradish peroxidase (MilliporeSigma Canada Ltd, Oakville, ON, Canada) at a dilution of 1:5000 were used. The detection of signals was carried out using SuperSignal™ West Pico PLUS Chemiluminescent Substrate (Fisher Scientific, Hampton, NH, USA).

## 3. Results

### 3.1. Vacuum-Based Agro-Infiltration Is the Best Approach for Agro-Infection

Four inoculation methods were tested and compared to assess their efficacy in launching the infection of grapevine plantlets using agrobacteria containing pLR3 (summarized in Table 1). The vacuum-based method gave the highest levels of infection, followed by pricking, while agro-drenching and injection failed to produce infection. A total of 39 of the 50 Syrah plantlets survived the vacuum-based inoculation procedure, with 22 plants (56%) testing positive for GLRaV-3 at 6 months post-inoculation (Table 1). For Cabernet franc, 38 of the 50 plantlets survived, and 20 (53% of survivors) tested positive for GLRaV-3 at 6 mpi. On the other hand, inoculation via the pricking method yielded a higher rate of survival but a much lower rate of infection. For example, 43 of the 50 Syrah plantlets survived, with only 7 testing positive (16% of survivors). Similarly, 42 of the 50 Cabernet franc plantlets survived, and only 6 of them (14% of survivors) were infected with GLRaV-3 (Table 1).

### 3.2. Impacts of Age and the Cultivar of Grapevine Plantlets on Survival and Infectivity Rates

Both the age and specific cultivars of the grapevine plantlets had considerable impacts on survival and infection rates. The survival rate was positively correlated with the age of plantlets (Table 2). For example, in the 5–7-weeks-old group, 18–25% of the plantlets survived after agro-infiltration; in the 8–11-weeks-old group, 65–82% of plantlets survived; while in the 12–16-weeks-old group, the survival rate reached 76% for Chardonnay, 88% for Cabernet franc, and 92% for Syrah. Based on the differences observed in the survival rates between the three cultivars, Syrah had the highest survival rate for all three age groups, and Chardonnay had the lowest (Table 2). On the other hand, the infectivity rate did not follow the same trend as the survival rate. The highest infection rate was consistently observed for plantlets in the 8–11-weeks-old group for all three cultivars. It is important to note that the infection rate varied considerably between the three cultivars, with Syrah having the highest at 63%, followed by Cabernet franc at 58%, and Chardonnay with the lowest at 44% (Table 2). In summary, when both the survival and infection rates are considered, 8–11-weeks-old grapevine plantlets gave the best performance for all three cultivars compared with the other two age groups. Syrah gave the best outcome in both survival and infection rates, followed closely by Cabernet franc, while Chardonnay ranked last. 

### 3.3. The Effects of Humidity Control on Plantlet Survival and Infectivity

The results from an initial trial of agro-infiltration showed that low humidity levels had drastic negative effects on the survival rate and, consequently, on the infection rate. We set up an experiment to test the effects of the duration of covering the newly infiltrated plantlets with a plastic sheet on the rates of survival and infectivity. As shown in Table 3, if the plastic covers were removed at one or two weeks post-infiltration (wpi), almost none of the plantlets survived. However, if the removals were performed step by step by poking the covers after the second week post-infiltration, the number of survivors and, consequently, the infection rate increased. Therefore, in subsequent experiments, all agro-infiltrated plantlets were kept under high relative humidity conditions for at least 3 weeks.

### 3.4. Effects of Vacuum Treatment Duration and Density of Agrobacterium on Infectivity Rates

To find out the optimal duration of vacuum application for survival and infection, three different experimental series were tested. These included three cycles of vacuum treatment for 5, 10, and 15 min, followed by a quick vacuum release for each cycle. The survival rate at 4 mpi was negatively correlated with the duration of vacuum treatment, as expected (Table 4). For instance, with the three 5 min cycles of vacuum application, 84% and 82% of Syrah and Cabernet franc plantlets survived, respectively, and in the case of using three cycles of 10 min vacuum treatment, 78% and 74% of Syrah and Cabernet franc plantlets survived, respectively. The lowest survival rate was observed in plantlets that were subjected to three cycles of 15 min treatment, where only 42% of Syrah plantlets and 36% of the Cabernet franc plantlets were viable (Table 4). Interestingly, the infection rate did not follow the same trend. The three 10 min vacuum cycles gave the best results, with 66% of the infiltrated Syrah plantlets and 64% of the Cabernet franc plantlets being positive for GLRaV-3. We thus concluded that the best vacuum cycle to initiate the viral infection of grapevine plantlets derived from tissue culture is three 10 min vacuum cycles.

The influences of the cell densities of the agrobacteria containing cDNA clones of GLRaV-3 and those containing the plasmid for the RSS p24 on the survival and infection rates were also tested. The best cell density or OD_600_ for the agrobacterium containing pLR3 was 2.0 regardless of whether p24 was used at either an OD_600_ of 0.5 or 1.0 (Table 5). All other combinations gave a lower percentage of infection. 

### 3.5. Effects of Co-Infiltration with Virus RSSs on Infectivity

In a preliminary experiment, it was found that co-infiltration with p24, an RSS encoded by GLRaV-2, was essential for launching infection with pLR3. To find out the most effective RSS for this purpose, the following five RSSs were tested in co-infiltration experiments: HC-Pro, p19, p21, TCV-CP, and p24. As negative controls, grapevine plantlets were infiltrated with only pLR3. All RSSs had major positive impacts on the infection rate, albeit to different degrees and depending on the cultivars used (Table 6). For example, p19 increased the rate of infection by 3.7-fold for Syrah and 2.6-fold for Cabernet franc compared with the no-RSS control. For both cultivars, the top three RSSs were p19, HC-Pro, and TCV-CP. Interestingly, p24 ranked fourth for Syrah and tied in fourthplace with p21 where Cabernet franc was concerned (Table 7).

**Figure 5:**
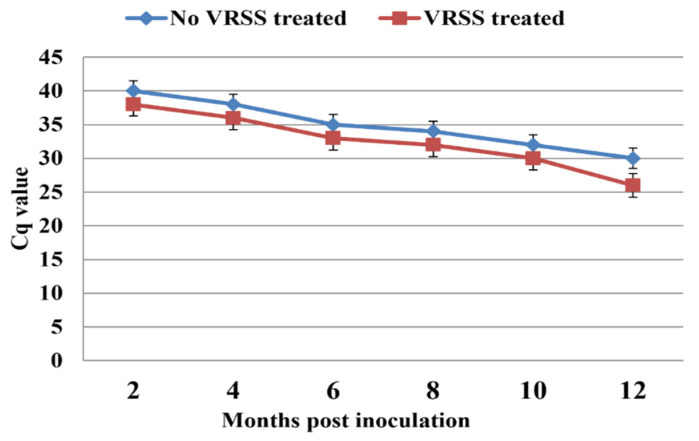
Effects of RNA-silencing suppressor (p24) on infection rate and viral titer in grapevines after inoculation with GLRaV-3 full-length clone via agro-infiltration. Results of RT-qPCR using primers F1-14117 and R-14327 for grapevines infiltrated with pLR3 (blue line) or co-infiltrated with pLR3 and RNA-silencing suppressor p24 (red line).

To test if the increase in the infection rate of grapevine plantlets in the presence of RSSs is due to enhanced virus replication, a time-course RT-qPCR analysis was conducted for three Syrah plants that were co-infiltrated with pLR3 and p24 at 2, 4, 6, 8, 10, and 12 mpi (Figure 5). It should be noted that the Cq values for the no-RSS-treated samples were consistently higher than the corresponding values for the RSS-treated samples, reflecting the lower levels of virus RNA compared with the RSS-treated samples. Two conclusions can be drawn from these data: (1) A positive signal was first detected at 2 mpi for the grapevine samples that were co-infiltrated with pLR3 and the RSS and with a 2-month delay in the grapevine samples without using the RSS. (2) As time progressed, the Cq values decreased for both and the difference in the Cq between the two groups of plants increased considerably at 12 mpi.

### 3.6. Effects of Dormancy Treatment on Infection Rate

**Figure 6:**
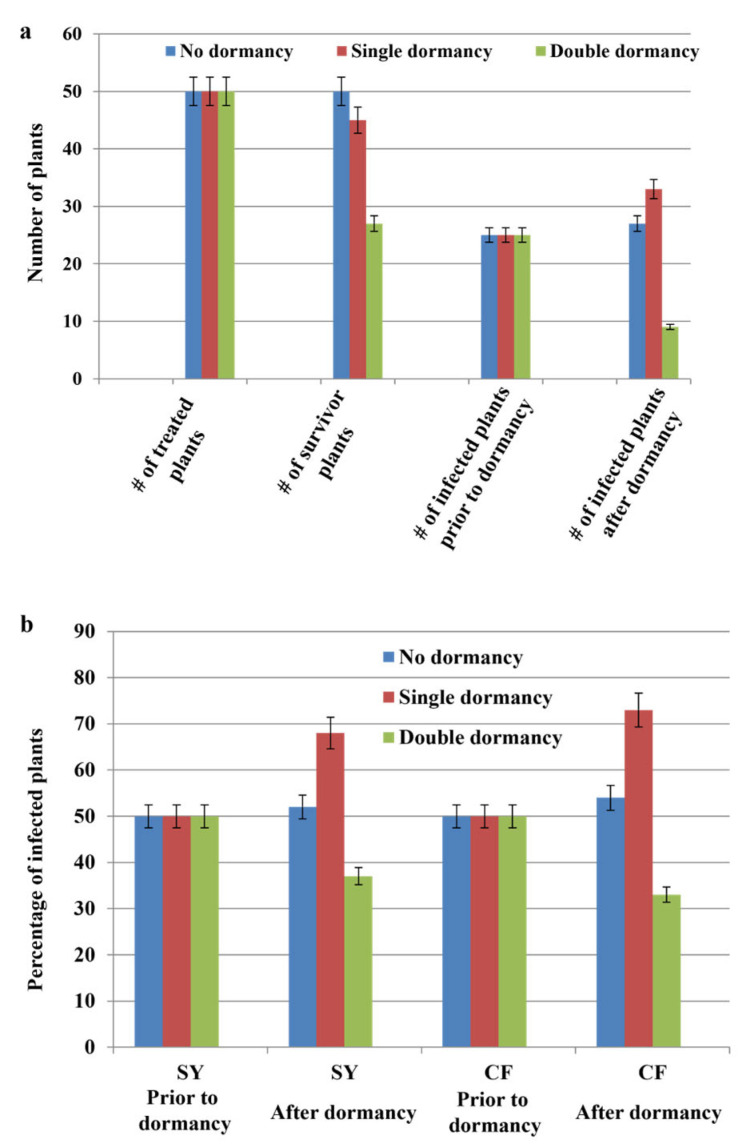
Effects of dormancy treatment on plantlet survival and infection rates. (**a**) The number of Cabernet franc plantlets that survived or tested positive for GLRaV-3 after undergoing a single cycle or two cycles of dormancy treatment at 4 °C compared with those in the no-dormancy-treatment group. (**b**) Percentage of Syrah or Cabernet franc plants that tested positive for GLRaV-3 prior to dormancy treatment compared with after dormancy treatment. Note: A single dormancy treatment resulted in a significant increase in infectivity rate, while double dormancy treatment decreased the percentage of plants infected by GLRaV-3.

The single-cycle dormancy treatment had a major positive impact on the infection rates in the infiltrated plantlets 12 months post-infection. For example, among the 47 Syrah plants that survived dormancy treatment, 32 tested positive for GLRaV-3, reflecting a 28% increase in the number of infected plants (Table 8). A similar trend was observed for the Cabernet franc plants: 33 of the 45 plants that survived the single-cycle dormancy treatment tested positive for GLRaV-3 (Table 8). In contrast, the double-cycle dormancy treatment was detrimental to plant survival, which, in turn, resulted in a reduction in the number of plants that tested positive for GLRaV-3 at the completion of the treatment cycle (Figure 6a,b). For example, 21 Syrah and 23 Cabernet franc plants out of 50 died due to undergoing two cycles of dormancy. Of the survivors, only 11 Syrah and 9 Cabernet franc plants tested positive for the virus (Table 8).

**Figure 7:**
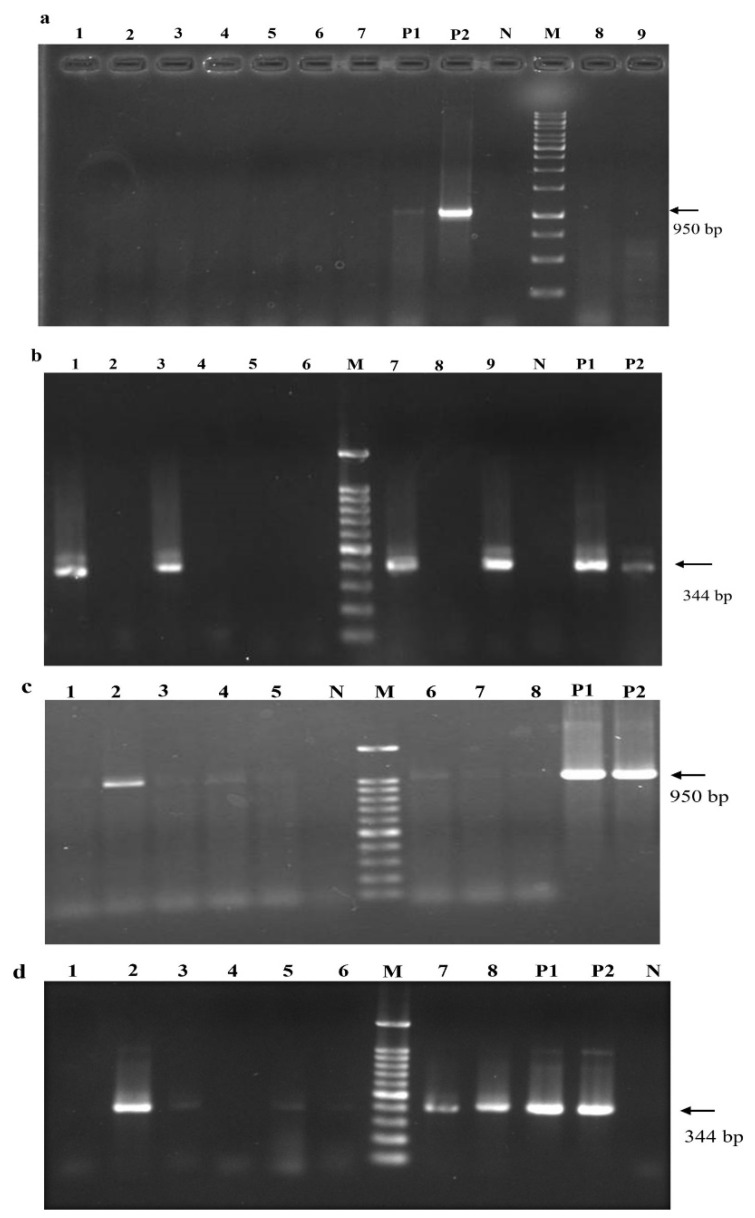
Effects of dormancy treatments on infection rate and viral titer in grapevines after inoculation with GLRaV-3 full-length clone via agro-infiltration. Results of first round of PCR amplification using external primers F1-16289 and R-17240 (**a**) and second round of PCR with internal primer set F1-16896 and R-17240 (**b**) at 4 mpi of grapevine plantlets infiltrated with both GLRaV-3 cDNA clone and p24 prior to dormancy. Lanes 1–9: samples collected from individual infiltrated grapevines. (**c**,**d**): Detection of GLRaV-3 using nested RT-PCR in grapevines that were co-infiltrated with GLRaV-3 full-length clone and p24. Grapevine plants were subjected to a single-cycle dormancy treatment. (**c**) Results of first round of RT-PCR using primers F1-16289 and R-17240. (**d**) Results of second round of PCR using primers F1-16896 and R-17240. M: molecular size marker; P1: agrobacterial cells containing GLRaV-3 full-length cDNA clone; P2: plasmid DNA containing pLR3; N: Cabernet franc vine mock-infiltrated with buffer; lanes 1–8: samples collected from individual infiltrated grapevines of Cabernet franc at 12 mpi of grapevine plantlets infiltrated with both GLRV-3 cDNA clone and p24 and after dormancy.

Although systemic infection was detectable after 4 mpi using nested RT-PCR and RT-qPCR, a single round of PCR amplification failed to detect any positives prior to dormancy treatment (Figure 7a,b). In contrast, after one dormancy cycle, even a single round of RT-PCR was able to identify GLRaV-3 positives. As shown in (Figure 7c,d), the titer of the virus at 12 mpi was high enough for some of the samples to test positive after the first round of nested RT-PCR. The intensity of PCR products further increased for those ones tested positive in the first round or became visible for those ones did not show positive amplification in the first round of PCR. 

**Figure 8:**
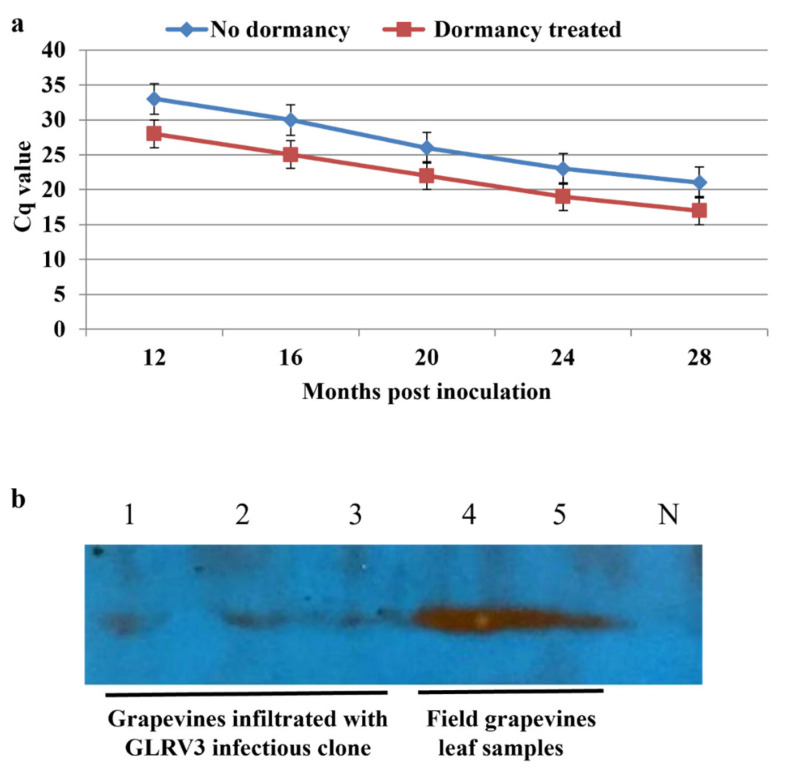
(**a**) Trends in GLRaV-3 titer judged using RT-qPCR testing of grapevines co-infiltrated with viral infectious clone and p24 that were subjected to a single-cycle dormancy treatment (red line) compared with those that did not undergo dormancy treatment (blue line). Primers F1-14117 and R-14327 were used in RT-qPCR. Note that at 12 mpi, the titer of GLRaV-3 was significantly higher after two months of dormancy. The results shown represent 3 biological replicates with 3 technical replicates for each sample. (**b**) The results of Western blotting for the capsid protein in leaf samples of Cabernet franc grapevines. Lanes 1, 2, and 3: leaf samples from grapevines infiltrated with pLR3 full-length cDNA clone, which tested positive for GLRaV-3 in nested RT-PCR. Lanes 4 and 5: leaf samples collected in October from field grapevines infected with GLRaV-3. These field samples, which were previously used to study the seasonal dynamics of GLRaV-2 and GLRaV-3 [35], were included as positive controls. N: grapevine infiltrated with agrobacteria containing p24 as a negative control.

The RT-qPCR results also confirmed those obtained from nested RT-PCR, which revealed that at 4 mpi and before dormancy treatment, the Cq value was too high (35–38) to be confidently called positive. However, after a single dormancy cycle, the Cq value was reduced to 25–30 at 12 mpi, whereas the Cq values of the non-dormancy treatment group collected at the same time point were between 30 and 35. Taken together, these data demonstrate that dormancy had a considerable positive impact on the viral titer (Figure 8a). Furthermore, the viral titer continued to rise (i.e., the Cq value continued to drop) for both the dormancy treatment group and the control group (Figure 8a). No virus capsid protein was detected via Western blotting prior to the one-year mark in contrast with the field samples used as positive controls (Figure 8b, lanes 4–5). However, at 14 mpi and after the dormancy treatment, the capsid protein could be confidently detected in vines infiltrated with pLR3 (Figure 8b, lanes 1–3).

## 4. Discussion

The state of research on viruses of woody perennial plants including grapevine lags far behind the research on those that infect herbaceous plants due to the compound effect of several factors. First, grapevines are commonly infected with a mixture of distinct viruses and strains, complicating the elucidation of the etiological contribution of individual viruses in a disease. Second, many pathogenic viruses of woody plants do not infect herbaceous plants, which are incomparably more amenable to investigating all aspects of viral infections. In this respect, GLRaV-2 seems to be a rare exception in being readily inoculated into *Nicotiana benthamiana* plants [36]. Third, unlike most viruses of herbaceous plants, grapevine viruses cannot be transmitted between vines via mechanical inoculation, although in some studies, dodders (*Cuscuta* spp.) have shown different levels of success in transferring some viruses to woody plants [37,38]. Therefore, the preferred way to initiate infection with a virus in grapevines is via the delivery of infectious clones using agrobacterium. This procedure, dubbed ‘agro-infection’, was invented by Grimsley et al. [39] to launch the infection of turnip with the *Cauliflower mosaic virus*. Since then, agrobacterium-based inoculation has been widely used in the plant virology community. In fact, this approach has become the method of choice when it comes to studies on viruses that infect grapevines and other woody perennials.

Drenching was the first agrobacterium-based inoculation method that was developed to launch grapevine infection using micro-propagated plantlets with an infectious DNA clone of GVA [20]. This method appears to be effective in launching grapevine infection with GVA and *Grapevine Pinot gris virus* cDNA clones [20,26]. Unfortunately, it failed to initiate systemic infection for GRSPaV at a practically acceptable level [22].

In 2012, a breakthrough was achieved through the development of a vacuum-based agro-infiltration method to launch the infection of micro-propagated grapevine plantlets with a GFP-tagged cDNA clone of GLRaV-2 [21]. The authors tested 15 grape cultivars and found that Syrah and Cabernet franc were most susceptible, and Zinfandel less so, whereas the remaining 12 cultivars failed to be infected. It was also shown that p24, an RNAi suppressor encoded by GLRaV-2, significantly improved the infection rate [21,40], opening the possibility of the method’s application for other grapevine-infecting viruses. The utility of this new technology was tested by Yepes et al. [24] to launch infection with an infectious clone of GRBV, a single-stranded DNA virus of the family *Geminiviridae* responsible for an emerging grapevine red blotch disease [41]. In the latter study, the effectiveness of vacuum infiltration in launching GRBV infection in grapevine plantlets derived from seven *V. vinifera* cultivars and four rootstocks was tested. It was concluded that(1) both vacuum agro-infiltration and agro-pricking worked equally well; (2) 4–6-week-old plantlets were superior to 8–12-week-old plantlets; (3) none of the three silencing suppressors (GLRaV-2 p24, TBSV p19, and CMV 2b) were necessary for initiating GRBV infection; and (4) the vacuum duration and level were specific to the grapevine cultivars tested [24]. The achieved rate of infection varied significantly among the grape cultivars: Syrah performed the best, with an average of 55% of the plantlets testing positive for GRBV, followed by Pinot noir (22%), and Cabernet franc and Chardonnay (20%). In contrast, the infection rates in rootstocks were lower, averaging at 16% among the four rootstocks [24].

In this study, we used a systematic approach to test a set of factors that may influence the survival and infection rate of grapevine plantlets. First, four inoculation methods for the grapevine plantlets with a cDNA clone of GLRaV-3 (unpublished data) were applied. Among these, the vacuum agro-infiltration method was the most effective, followed by pricking, whereas soil drenching and direct infiltration with a needleless syringe failed to produce infection. We demonstrated that plantlets of 8–11 weeks of age gave the best performance both in terms of survival and infectivity. We compared five different silencing suppressors in agro-infiltration and demonstrated that four of them were highly effective as each substantially increased the infection rate by 40–50%. In line with previous research [21,24], maintaining plantlets under high humidity during the first several weeks after infiltration was critical for the plantlets’ survival and, hence, infectivity. Lastly, we showed that a single cycle of dormancy treatment for two months at 4 °C significantly increased the viral titer and enhanced symptom development.

It was also reconfirmed that the grapevine cultivar and the age of plantlets have direct impacts on survival and infection rates. The 8–11-week-old plantlets showed the best outcomes in survival (65–82%) and infectivity (44–63%) rates, while the older and younger plantlets resulted in either reduced infectivity or survival rates similar to the findings of Yepes et al. [24]. Also consistent with the reports by Kurth et al. [21] and Yepes et al. [24], Syrah and Cabernet franc showed higher survival and infection rates compared with Chardonnay.

We also found that the application of virus RNA-silencing suppressors was an important factor in the virus infection rates and viral titer, in agreement with the results of Kurth et al. [21] but contrasting with the findings of Yepes et al. [24]. This difference could be due to differences in genome type, genome size and structure, expression strategies, and infection processes between GRBV and GLRaV-3. Indeed, GRBV possesses a single-strand DNA genome of only ~3.2 kb, whereas GLRaV-3 has one of the largest positive-sense ssRNA of nearly 19 kb.

As mentioned above, the GLRaV-3 titer and infection rates exhibited a sharp variation before and after cold treatment. According to the RT-qPCR and nested-RT-PCR analyses of the samples collected prior to and after dormancy treatment, the cold treatment increased the titer and infection rates by16% and 19% in Syrah and Cabernet franc, respectively, similar to the findings of Yepes et al. [24] with an infectious clone of GRBV.

## 5. Conclusions

To our knowledge, this is the most comprehensive report on the optimization of agro-inoculation methods for launching grapevine infections with a viral cDNA clone. In particular, we established that the best material for inoculation is 2–4-month-old micro-propagated Syrah or Cabernet franc plantlets. We also found that co-infiltration of the plantlets with a strong suppressor of RNA silencing, such as HC-Pro, p19, or p24, significantly increases the infection rate. Upon agro-infiltration, plantlets must be protected from loss of humidity during the first 3 weeks. Finally, we showed that a single cycle of dormancy treatment of lignified plantlets at 4 °C for 2 months significantly increases the viral titer.

This work is certain to facilitate overall progress in the field of grapevine virology by providing an optimized protocol for grapevine agro-infection. This protocol constitutes a critical tool for investigations into the etiological roles of distinct viruses in grapevine disease complexes often caused by several viruses, as well as advanced studies on the molecular and cellular aspects of virus–host interactions in grapevines.Furthermore, the methodical approaches developed in this work have clear potential applications for studying challenging but important pathogenic viruses of other woody crop plants such as citrus, stone fruits, and pome trees.

## Figures and Tables

**Table 1:** Effects of different agro-infiltration methods on grapevine plantlet survival and infection rates. The status of infection was based on nested RT-PCR at 6 months post-inoculation (mpi). SY: Syrah; CF: Cabernet franc.

Inoculation Method	Vacuum-Based Agro-Infiltration		Agro-Pricking		Agro-Drenching		Agro-Injection	
Cultivar of Plantlets	SY	CF	SY	CF	SY	CF	SY	CF
No. of infiltrated plantlets	50	50	50	50	50	50	50	50
No. of survivor plantlets	39	38	43	42	46	45	44	43
No. of infected plantlets	22	20	7	6	0	0	0	0
Percentage of infection of survivor plantlets	56%	52%	16%	14%	0%	0%	0%	0%

**Table 2:** Effects of age and cultivars of grapevine plantlets on survival and infection rates. The status of infection was based on nested RT-PCR at 4 mpi. SY: Syrah; CF: Cabernet franc; CH: Chardonnay.

Age of Plantlets	5–7 Weeks Old			8–11 Weeks Old			12–16 Weeks Old		
Cultivar of Plantlets	SY	CF	CH	SY	CF	CH	SY	CF	CH
No. of infiltrated plantlets	100	100	100	100	100	100	100	100	100
No. of survivor plantlets	25	21	18	82	78	65	92	88	76
No. of infected plantlets	14	11	8	52	46	29	13	10	5
Percentage of infection of survivor plantlets	56%	52%	44%	63%	58%	44%	14%	11%	7%

**Table 3:** Effects of humidity control on survival and infection rates of Syrah plantlets after vacuum-based agro-infiltration. Status of infection was assessed using nested RT-PCR at 4 mpi.

Time of Cover Removal	1 Week		2 Weeks		3 Weeks	
Manner of Cover Removal	Instant	Gradual	Instant	Gradual	Instant	Gradual
No. of infiltrated plantlets	100	100	100	100	100	100
No. of survivor plantlets	0	6	2	18	36	78
No. of infected plantlets	0	0	0	4	11	51
Percentage of infection of survivor plantlets	0%	0%	0%	22%	30%	65%

**Table 4:** Effects of vacuum duration on survival and infection rates. Grapevine plantlets that were co-infiltrated with agrobacterium containing pLR3 and the suppressor of RNA silencing p24 were tested using nested RT-PCR at 4 mpi for the presence of GLRaV-3.

Vacuum Duration	5 min		10 min		15 min	
Cultivar of Plantlets	SY	CF	SY	CF	SY	CF
No. of infiltrated plantlets	50	50	50	50	50	50
No. of survivorplantlets	42	41	39	37	21	18
No. of infected plantlets	8	5	26	24	12	11
Percentage of infection of survivor plantlets	19%	12%	66%	64%	57%	61%

**Table 5:** The effects of agrobacterial cell density (OD_600_) on infection and survival rates. Status of infection was based on nested RT-PCR at 4 mpi.

OD 600	GLRaV-3	1		2		3	
	P24	0.5	1	0.5	1	0.5	1
Cultivar of Plantlets		SY	SY	SY	SY	SY	SY
No. of infiltrated plantlets		50	50	50	50	50	50
No. of survivor plantlets		42	39	38	34	23	18
No. of infected plantlets		5	9	26	24	15	11
Percentage of infection of survivor plantlets		12%	23%	68%	70%	65%	61%

**Table 6:** Effects of co-infiltration with RNA-silencing suppressors on infectivity percentage. Grapevine plantlets aged 8–11 weeks were infiltrated with agrobacterium containing infectious pLR3 or pLR3 and one of the following RNA-silencing suppressors: HC-Pro, p19, p24, TCV CP, or p21. The status of GLRaV-3 infection was assessed based on nested RT-PCR at 4 mpi.

RNA-Silencing Suppressors	No SRS		HC-Pro		P24		P19		TCV		P21	
	SY	CF	SY	CF	SY	CF	SY	CF	SY	CF	SY	CF
No. of infiltrated vines	50	50	50	50	50	50	50	50	50	50	50	50
No. of survivor vines	44	40	42	37	40	39	44	38	39	36	43	38
No. of infected vines	8	9	22	19	20	18	26	23	21	23	17	18
Percentage of infection of survivor vines	18%	23%	52%	51%	50%	51%	59%	60%	54%	64%	40%	47%

**Table 7:** Ranking of different RNA-silencing suppressors based on the impacts of them on the infectivity percentage.

Rank	Based on Infection % of All Plants		Based on Infection % of Survivor Plants	
	SY	CF	SY	CF
1	P19 (52%)	TCV (46%)	P19 (59%)	TCV (64%)
2	HC-Pro (44%)	P19 (46%)	TCV (54%)	P19 (60%)
3	TCV (42%)	HC-Pro (38%)	HC-Pro (52%)	HC-Pro (51%)
4	P24 (40%)	P24 (36%)	P24 (50%)	P24 (46%)
5	P21 (34%)	P21 (36%)	P21 (40%)	P21 (47%)
No RSS	16%	18%	18%	23%

**Table 8:** Effects of dormancy treatment on survival and infection rates of grapevine plantlets that were infiltrated with pLR3. Plantlets were subjected to either a single dormancy treatment for two months or two cycles of dormancy treatment for two months each. Status of GLRaV-3 infection was assessed using nested RT-PCR.

Duration of Dormancy at 4 °C	No Dormancy Treatment		Single Dormancy		Double Dormancy	
Cultivar of Plantlets	SY	CF	SY	CF	SY	CF
Total no. of infiltrated plantlets	50	50	50	50	50	50
No. of plants that survived dormancy treatment	50	50	47	45	29	27
No. of infected plantlets prior to dormancy	25	25	25	25	25	25
No. of plantlets that tested positive after dormancy	26	27	32	33	11	9
Percentage of infection of survivor plantlets	52%	54%	68%	73%	37%	33%

## Data Availability

Data is contained within this article and Supplementary Materials.

## Supplementary Materials

The following supporting information can be downloaded at: https://www.mdpi.com/article/10.3390/pathogens12111314/s1, Table S1. Agar media for all grape stages OH, GS1, GS2 and GS3. Table S2. Primers were used in this study.
