# Supplementary material for: Optimization of a Protocol for Launching Grapevine Infection with the Biologically Active cDNA Clones of a Virus
Source: Pathogens. 2023 Nov 3;12(11):1314. doi: 10.3390/pathogens12111314 (PMC10674828; doi:10.3390/pathogens12111314)
Supplement: Supplementary file 1 [file pathogens-12-01314-s001.zip › pathogens-2675069-supplementary.pdf]

pathogens

MDPI

Article

Optimization of a Protocol for Launching Grapevine Infection with the Biologically Active cDNA Clones of a Virus

Mehdi Shabanian[1,*], Caihong Li [1], Ali Ebadi [2], Valerian Dolja[3] and Baozhong Meng [1]

[1] Department of Molecular and Cellular Biology, University of Guelph, Guelph, ON N1G 2W1, Canada; caihong@uoguelph.ca (C.L.); bmeng@uoguelph.ca (B.M.)
[2] Department of Horticulture, College of Agriculture and Natural Resources, University of Tehran, Karaj 31587–11167, Iran; aebadi@ut.ac.ir
[3] Department of Botany and Plant Pathology, Oregon State University, Corvallis, OR 97331, USA; doljav@oregonstate.edu
* Correspondence: shabania@uoguelph.ca

Supplemental Tables:

Citation: Shabanian, M.; Li, C.; Ebadi, A.; Dolja, V.; Meng, B. Optimization of a Protocol for Launching Grapevine Infection with the Biologically Active cDNA Clones of a Virus. *Pathogens* **2023**, *12*, 1314. https://doi.org/10.3390/pathogens12111314

Academic Editors: Kathleen Hefferon and Srividhya Venkataraman

Received: 6 October 2023
Revised: 25 October 2023
Accepted: 30 October 2023
Published: 3 November 2023

Copyright: © 2023 by the authors. Submitted for possible open access publication under the terms and conditions of the Creative Commons Attribution (CC BY) license (https://creativecommons.org/licenses/by/4.0/).

Table S1. Agar media for all grape stages OH, GS1, GS2 and GS3.

**Media Stage**<br>Ingredients/1 L	**OH medium.**<br>**pH: 5.7**	**GSI**<br>**pH: 5.3**	**GS2**<br>**pH: 5.3**	**GS3**<br>**pH: 5.3**
M & S salts	3.22 g	3.22 g	3.22 g	3.22 g
Thiamine	0.8 mL (0.5 mg/mL)	0.8 mL (0.5 mg/mL)	0.8 mL (0.5 mg/mL)	0.8 mL (0.5 mg/mL)
Inositol	0.100 g	0.1 g	0.025 g	0.025 g
Sucrose	7 g	20 g	15 g	12.5 g
BAP	_	2 mL (0.5 mg/mL)	4 mL (0.5 mg/mL)	_
NaH_2PO_4	0.170 g	_	0.05 g	_
Adenine sulfate	0.08 g	_	_	_
IAA	_	_	0.5 mL (1mg/mL)	1 mL (1mg/mL)
Agar	3 g	2 g	2 g	1 g
Gelrite	_	1.13 g	1.2 g	1 g
Cefotaxime	0.2 g	0.2 g	0.2 g	0.2 g

Table S2. Primers were used in this study.

**Primers**	**Sequences**	**Amplicon (bp)**	**Target Gene**
LR3CP107F	5′-TCTTAAARTAYGTTAAGGACGG-3′	836	CP of GLRaV-3
LR3CP942R	5′-CTAGTTCTTTTGCAATAGTTG-3′		
LR3_14117F	5′-GTGCCGGAATCTGAGACTCT-3′	211	
LR3_14327R	5′-GCCCATAACCTTCTTACACAGC-3′		
LR3_16289F	5′-GTTCGGAGACGTATAACATC-3′	951	ORF8 &9 of GLRaV-3
LR3_17240R	5′-CTAATCCTCGATTCTCCCCTGAC-3′		
LR3_16896F	5′-CGCTTAAAACGAACGATCAC-3′	344	ORF9 of GLRaV-3
LR3_17240R	5′-CTAATCCTCGATTCTCCCCTGAC-3′		
LR2-C-F	5′-AAGGAATACTTAGGCGCCGAC-3′	1458	3′ end region including GFP of GFP-tagged GLRaV-2
LR2-C-R	5′-AGGAGCCCCTTTCTGCACCAA-3′		
eGFP-F	5′-AAAGGTACCGTGAGCAAGGGCGAGGA-3′	720	eGFP
eGFP-R	5′-AAAGGATCCTTACTTGTCAGCTCGTCCATGC-3′		
ACT-1021F	5′-ATCAGGAAGGACCTCTATGG-3′	206	Actin 1 of grapevine
ACT-1226R	5′-ATCCACATCTGCTGGAAGG-3′		
